# Case report: Heterotopic intrarenally located adrenocortical oncocytoma

**DOI:** 10.12688/f1000research.3780.1

**Published:** 2014-03-17

**Authors:** Konstantin Godin, Nicole Bang, Yuri Tolkach

**Affiliations:** 1 Südharz Klinikum Nordhausen gGmbH, Urology Clinic, Nordhausen, 99734, Germany; 2 Südharz Klinikum Nordhausen gGmbH, Pathology Clinic, Nordhausen, 99734, Germany; 3 Medizinische Hochschule Hannover, Urology and Urologic Oncology Clinic, Hannover, 30625, Germany

## Abstract

The clinical case of a 65-year-old woman with an incidentally detected left-sided mass in the upper renal pole is presented. A functional adrenal tumor was excluded. The mass was removed retroperitoneoscopically. The perioperative period was uneventful. The histopathological examination revealed a heterotopic intrarenal adrenocortical oncocytoma.

Adrenal oncocytic neoplasms are very rare, with, to the authors’ knowledge, only 159 described cases so far. Most cases are non-functioning adenomas that can reach a considerable size. Only 10 heterotopic adrenal oncocytomas have been described (three retroperitoneal and seven intraspinal cases). Although the intrarenal adrenal rest is the most frequently appearing variant of adrenal heterotopia, to the best of our knowledge, this report is the first description of an intrarenally growing adrenocortical oncocytic adenoma.

In addition to retroperitoneally located oncocytomas, this case could be interesting for urological practice because there are no diagnostic features which could provide a secure preoperative diagnosis of an adrenal oncocytic neoplasm and its malignant variant. Generally accepted indications for surgery of adrenal masses have to be respected. The definitive pathologic diagnosis is in most cases surprising because of its rarity. Benign adrenal oncocytic neoplasms do not require any adjuvant treatment. The oncocytic variant of adrenocortical carcinoma generally has a poor prognosis.

## Case report

**Figure 1.**
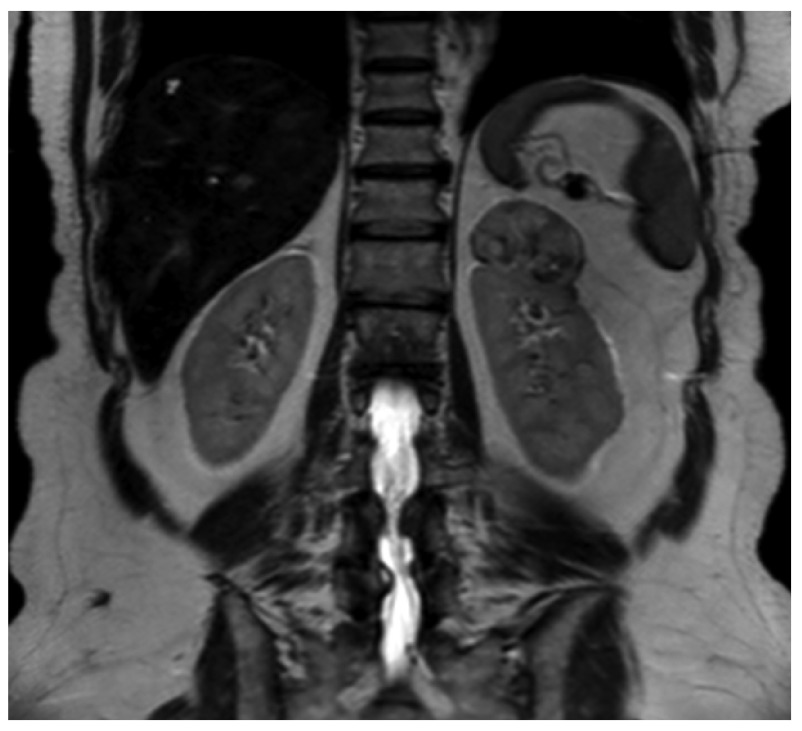
Mass in the left upper renal pole (T2-weighted MR image) with a size of 48×48×33 mm having a diffusely heterogeneous structure.

A 65-year-old Caucasian woman with a history of arterial hypertension underwent an ultrasound examination of the kidneys performed by her general practitioner in 2012. A left-sided suprarenal mass measuring about 5 cm was detected. The physical examination showed no palpable masses in the abdomen, and the peripheral lymph nodes were not enlarged. A 3-T magnetic resonance imaging (MRI) of the adrenal glands presented a heterogeneously enhancing mass measuring 48×48×33 mm in the left apical renal pole in contact with the adrenal gland ( Figure 1). The findings seemed to suggest the presence of a renal cell carcinoma. A differential diagnosis of pheochromocytoma had also been considered. Enlarged intra- or retroperitoneal lymph nodes were not detectable in the MRI. Catecholamines and metanephrines, aldosterone-renin ratio and serum cortisol before and after inhibition were within normal range in a 24 hour-urine sample analysis. Thus, a functional adrenal tumor could be excluded.

**Figure 2.**
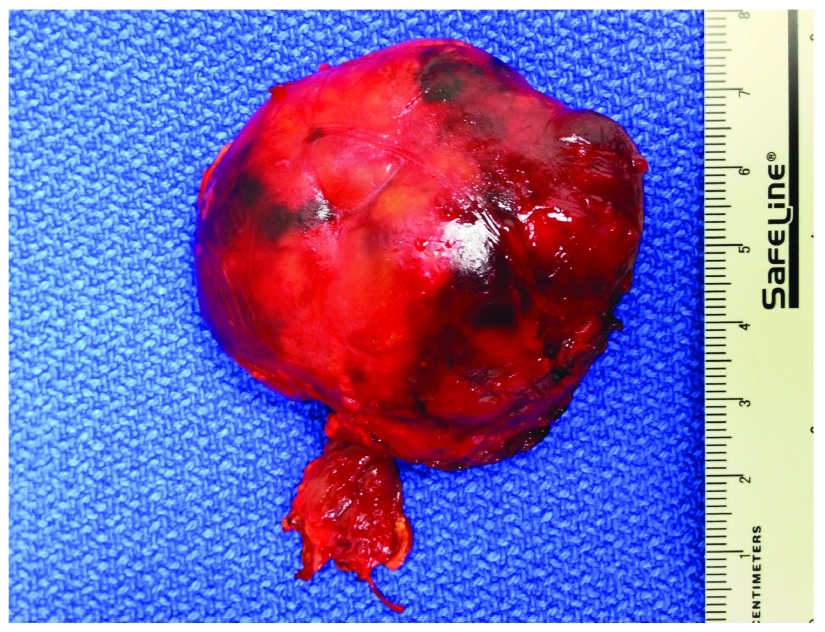
Specimen with adjacent part of left adrenal gland after retroperitoneoscopically performed tumor enucleation.

**Figure 3.**
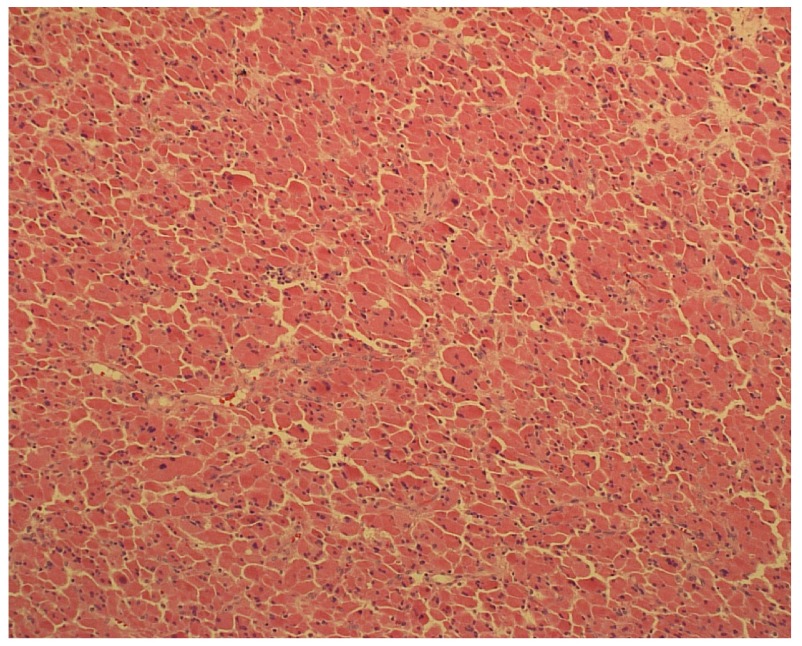
Histopathology section showing diffuse proliferation of oncocytes (Haematoxyline-eosin staining, magnification 200×).

The patient underwent a retroperitoneoscopic exploration. A round shaped 4.5 cm exophytic mass of the upper renal pole was excised retroperitoneoscopically in the fashion of a renal mass enucleation applying the zero-ischemia technique. Intraoperatively, the mass had a very thin, poorly defined pseudo-capsule which was adhering to the renal parenchyma caudally and to the macroscopically inconspicuous adrenal gland medially. The adrenal gland was removed *en bloc* with the mass ( Figure 2). No judgement could be made regarding the origin of the tumor. The postoperative recovery period was uneventful. Histopathological examination (haematoxyline-eosin) of the specimen revealed an oncocytic adrenocortical adenoma that arose from the heterotopic intrarenal adrenal tissue ( Figure 3).

The follow-up of this patient (approximately 2 years by the date of article submission) was uneventful. No additional treatment was necessary.

## Discussion

Oncocytic neoplasms of the adrenal glands are extremely rare. A PUBMED search up to December 2013 uncovered only 159 cases of adrenocortical oncocytomas since the first description of this tumor in adrenal in 1986 ^17^. Most of the papers describe only single patient case reports.

Oncocytic neoplasms or oncocytomas are mostly benign tumors which commonly occur in the kidneys, thyroid, parathyroid, pituitary and salivary glands ^1, 2^ and rarely in the respiratory tract ^3, 4^, choroid plexus ^5^ and adrenal glands. Oncocytic neoplasms of the adrenal glands, with, to the authors’ knowledge, only 159 previously described cases, are an extremely rare phenomenon. Most of these tumors were discovered as incidental findings on CT or MRI as non-functional adrenal masses. Various malignant potential can be determined in 20% of adrenocortical oncocytic neoplasms ^6^. Oncocytomas arising from the heterotopic adrenocortical tissue have been described in only 10 case reports, seven cases were located intraspinally ^7– 13^ and three cases in the retroperitoneum ^14– 16^. An oncocytic adenoma arising from heterotopic intrarenal adrenocortical tissue was not suspected in the preoperative assessment of this case because this entity has not been described previously. However, in addition to retroperitoneally located oncocytomas, this should be considered in the future as a differential diagnosis in cases presenting with an intrarenal or retroperitoneal mass.

This paper contains the first report on an oncocytic adenoma arising from the heterotopic intrarenal located adrenal tissue. Heterotopic adrenal tissue or adrenal rest presented mostly by cortical structures is more frequently located in the kidney. Other sites such as the celiac trunk, epididymis, spermatic cord, ovary, broad ligament, liver capsule, gallbladder, pancreas and spleen are rare ^18^.

Oncocytic neoplasms are mostly encapsulated masses with a brown or yellow surface on cut-section. The radial scar can be absent. Oncocytic neoplasms microscopically consist of so called oncocytes, large cells with rich eosinophilic granulations due to the high concentration of mitochondria. The Weiss criteria ^19^, which are commonly used in the histological diagnosis of adrenocortical malignancies, are not applicable to adrenocortical oncocytic neoplasms because all tumors have eosinophilic tumor cytoplasm, diffuse architecture and nuclear atypia. The modified Lin-Weiss-Bisceglia system differentiates between major, minor and definitional criteria for malignancy ^6^. None of these criteria is present in benign adrenal oncocytic neoplasms. Masses with uncertain malignant potential demonstrate the presence of one to four minor criteria (>10 cm or >200g, necrosis, capsular invasion or sinusoidal invasion) in absence of major criteria (mitotic rate >5 mitoses per 50 high-power fields, any atypical mitoses or venous invasion). In adrenal oncocytic carcinomas any of the major criteria could be present.

There are no specific criteria on both computed tomography and MRI and MRI with chemical shift subtraction for adrenal oncocytic neoplasm and its malignant variant ^20^. The bulk size cannot be used as a reliable criterion to estimate the risk of malignancy.

Eighty-three percent of adrenal oncocytic neoplasms are non-functioning masses ^21^. In rare cases, an adrenal oncocytic neoplasm can produce catecholamines, cortisol or testosterone ^21^.

The therapeutic standard is a minimally invasive adrenalectomy. In cases of a large mass, infiltration of surrounding structures, and lymph node bulks, an open surgery approach should be chosen.

Today, there are no recommendations for the follow-up of benign adrenal oncocytic neoplasms. Only one local recurrence of a neoplasm that was originally diagnosed as benign has been described and fulfilled the criteria of uncertain malignant potential ^21^. The oncocytic variant of adrenocortical carcinoma has a poor prognosis, with a postoperative recurrence rate of 75%, and a tumour-related mortality of 40% in a small group of 24 patients with a median follow-up of 21 months (range: 1 – 180 months) ^21^. An adjuvant or palliative chemotherapy with mitotane can be administered in patients with adrenocortical carcinomas with beneficial effects ^22^, but there is no evidence of efficacy when applied to oncocytic variant.

## Conclusion

To the authors’ knowledge, this case report presents the first description of a heterotopic intrarenally located adrenocortical oncocytoma. Although being a rare location, this case is worth mentioning, given the challenging situation with regard to the diagnostics and differentiation from the potentially aggressive malignant lesions. Taking in account the absence of radiological criteria of a benign character, this tumor should be considered and treated as a malignant lesion, although a minimally invasive approach should be chosen when possible. The definitive pathologic diagnosis is in most cases surprising because of the rarity of this type of neoplasm and radiological appearance mimicking renal cell carcinoma or adrenal carcinoma. Benign adrenal oncocytic neoplasms do not require any adjuvant treatment.

## Informed consent

Written informed consent for the publication of clinical details and clinical images was obtained from the patient.
