# Supplementary material for: Case report: Heterotopic intrarenally located adrenocortical oncocytoma
Source: F1000Res. 2014 Mar 17;3:73. [Version 1] doi: 10.12688/f1000research.3780.1 (PMC4168838; doi:10.12688/f1000research.3780.1)
Supplement: Peer review history

# Peer review: Referee response for version 1

**DOI:** 10.5256/f1000research.4049.r4152

**Published:** 2014-04-09

**Authors:** Levent Turkeri

**Affiliations:** 1 Department of Urology, Marmara University School of Medicine, Istanbul, Turkey

This is a report of a very interesting case of a heterotopic intrarenal adrenocortical oncocytoma.

As the authors stated this may be the first reported case in the literature.

The major concern is the appearance of the mass in relation to the kidney at figure 1, exactly at the location of left adrenal gland. The authors stated that they performed a retroperitoneoscopical enucleation and, intraoperatively, the mass had a very thin, poorly defined pseudo-capsule which was adhering to the renal parenchyma as well as to the macroscopically inconspicuous adrenal gland.

These observations raise the possibility that this mass was in fact arising from the adrenal gland itself and not from a heterotopic location within the kidney. A tumor of 4.5 cm located intrarenally would be difficult to enucleate without interfering with any renal parenchyma and therefore vascular structures - causing bleeding.

The provided histopathological figure displays only diffuse sheets of oncocytic cells without any evidence any surrounding renal tissue.

I would recommend a repeat discussion with the pathologist to see if this not really an adrenal oncocytoma, which would be still a rare entity, albeit with more than 50 cases reported in the literature.

I have read this submission. I believe that I have an appropriate level of expertise to confirm that it is of an acceptable scientific standard, however I have significant reservations, as outlined above.

# Response

**Published:** 2014-05-07

**Authors:** Yuri Tolkach

**Affiliations:** Urology Clinic, Military Medical Academy, Russian Federation

Dear Professor Turkeri,

Thank you for your evaluation.

Indeed, this tumor arose from the renal parenchyma and in fact left adrenal was intact and had no continuous contact to the tumor.

We have discussed your concerns with the pathologist and in the revised version of the article we will provide a pathology slice, where the nearby renal parenchyma and normal adrenal cells could be seen direct by the tumor, making the final diagnosis undoubtful.

With best wishes,

Konstantin Goding, Nicole Bang and Yuri Tolkach

# Peer review: Referee response for version 1

**DOI:** 10.5256/f1000research.4049.r4146

**Published:** 2014-04-07

**Authors:** M Hammad Ather

**Affiliations:** 1 Department of Surgery, Aga Khan University, Karachi, 74800, Pakistan

The authors report a heterotopic adrenal oncocytoma in a 65 year-old lady with essential hypertension. A routine ultrasound detected a 5 cm adrenal mass, however, MRI showed a 4.8 x 4.8 x 3.3 cm mixed intensity upper polar renal mass. Features were not suggestive of a malignant tumour. Urinary and blood work up excluded a functioning adrenal adenoma. The oncocytoma was removed retroperitoneoscopically with the zero-ischemia technique.

It is a well written report, however, I have the following observations on the current submission:

1. The sequence of the work up is not clear: was the MRI done first; or were urinary catecholamines and metanephrines, aldosterone-renin ratio and serum cortisol determined first? In the latter case, why was a pheochromocytoma suspected when neither the clinical manifestations nor imaging (MR) suggested a pheochromocytoma?
2. The authors have rightly noted that the imaging characteristics of benign and malignant oncocytoma are not clearly described in the current literature - they were entertaining a diagnosis of an RCC pre-operative. Did the authors consider doing a biopsy prior to surgical intervention?  It would be pertinent to mention indications for biopsy in such a situation, and discuss its merits and demerits. 

I have read this submission. I believe that I have an appropriate level of expertise to confirm that it is of an acceptable scientific standard, however I have significant reservations, as outlined above.

# Response

**Published:** 2014-05-07

**Authors:** Yuri Tolkach

**Affiliations:** Urology Clinic, Military Medical Academy, Russian Federation

Dear Dr. Ather,

Thank you for you valuable comments.

With regard to the first point: hormonal evaluation was done after MRI investigation to exclude the probability of hormone-producing tumor, while the tumor was located very close to the adrenal, though the probability was indeed very small. It was done according to the local guidelines.

Concerning the second point, based on contemporary evidence it is hard to discuss the necessity of biopsy both in adrenal and renal tumors. We did not consider this diagnostic modality. In our opinion, in this case there was no place for the biopsy.

With kind regards,

Konstantin Godin, Nicole Bang and Yuri Tolkach
